# Novel strategies to prevent and overcome relapse after allogeneic hematopoietic cell transplantation in acute lymphoblastic leukemia

**DOI:** 10.3389/fimmu.2023.1191912

**Published:** 2023-06-08

**Authors:** Mohammad Hassan Hodroj, Iman Abou Dalle, Nour Moukalled, Jean El Cheikh, Mohamad Mohty, Ali Bazarbachi

**Affiliations:** ^1^ Division of Hematology & Oncology, Department of Internal Medicine, American University of Beirut Medical Center, Beirut, Lebanon; ^2^ Bone Marrow Transplantation Program, American University of Beirut Medical Center, Beirut, Lebanon; ^3^ Sorbonne University, Saint-Antoine Hospital, AP-HP, INSERM UMRs 938, Paris, France

**Keywords:** acute lymphoblastic leukemia, allogeneic stem cell transplant, relapse, tyrosine kinase inhibitors, bispecific antibodies, antibody drug conjugates

## Abstract

The outcome of B-cell acute lymphoblastic leukemia (B-ALL) has improved over time with the incorporation of multi-agent chemotherapy in the treatment landscape as well as the recent approval of immunotherapeutic agents allowing a larger proportion of patients to undergo allogeneic hematopoietic cell transplantation (allo-HCT) which is still considered a potential curative approach. However, relapse post-transplant is still occurring and constitutes a common cause of treatment failure in B-ALL. The present review aims to discuss the novel strategies and therapies used to prevent and overcome relapse post allo-HCT in patients with ALL, focusing on the role of tyrosine kinase inhibitors in Philadelphia chromosome positive B-ALL, the role of innovative agents such as blinatumomab and inotuzumab ozogamicin, and finally the role of cellular therapy.

## 1 Introduction

During the last decade, the survival of adult patients with acute lymphoblastic leukemia (ALL) has significantly improved with the development of treatment strategies through the incorporation of targeted therapies such as tyrosine kinase inhibitors (TKI) (1), rituximab (2), novel immunotherapeutic agents (3), modification of consolidation and intensification courses based on the pediatric inspired protocols (4), and introduction of the concept of the minimal/measurable residual disease (MRD)-guided approach (5). Despite improvement of outcomes using novel agents, allogeneic hematopoietic cell transplantation (allo-HCT) is still considered a potentially curative approach in the management of high-risk ALL in first or second complete remission (CR) (6). Optimizing allo-HCT outcomes through conditioning regimens, graft-versus-host disease (GVHD) prophylaxis, supportive care, and post-transplant disease monitoring have led to favorable outcomes, however, relapses still occur. One of the well-established strategies to mitigate relapse is prophylactic and/or pre-emptive maintenance therapies post allo-HCT. Maintenance treatment could have a role in suppressing the leukemia clone, especially in the early post-transplant phase until a graft-versus-leukemia (GVL) effect is activated.

In line with what we previously studied in acute myeloid leukemia (AML), many transplant centers are implementing maintenance strategies either by pharmaceutical interventions such as FLT3 inhibitors in FLT3 mutant AML and hypomethylating agents, or by cellular therapies, mainly the use of donor lymphocyte infusion (DLI) (7–9). In ALL, other than immunosuppression tapering, and TKIs in Philadelphia chromosome (Ph) positive ALL, there are no known options for transplant physicians to maintain deep remissions. This current review aims to discuss the novel strategies and therapies used to prevent and overcome relapse post allo-HCT in patients with ALL (**Table 1**).

**Table 1:** Summary of the current strategies for prevention of relapse in ALL.

Prevention of Relapse in ALL	
Established Strategies	Innovative Strategies
**Tyrosine kinase inhibitors (TKIs)** The use of TKIs from all generations in Ph+ ALL patients as prophylactic therapy or preemptive therapy post allo-HCT has shown better leukemia- free survival and OS in prospective and retrospective studies (10)	**Blinatumomab** The use of blinatumomab maintenance post allo-HCT was associated with a cumulative incidence of relapse of 29%, 1-year OS of 85% and PFS of 71% (13) Blinatumumab is considered a promising agent to maintain remission and manage relapse post allo-HCT as a single agent or in combination.
**Donor Lymphocyte Infusion** The effectiveness of DLI use in relapsed ALL is still limited. DLI has been proven to reduce relapse risk and increase survival post allo-HCT if used in multiple escalated doses as prophylactic therapy or preemptive therapy post allo-HCT (11, 12)	**Inotuzumab Ozogamicin** A phase I trial showed that low dose maintenance inotuzumab ozogamicin post allo-HCT was well tolerated in high-risk ALL patients with absence of relapse in patients who completed one year follow up (14)
	**CAR T-cell Therapy** The data about CAR T-cell therapy for post allo-HCT relapse is scarce. Ding et al. showed satisfactory initial efficacy and safety of CAR T-cell therapy especially autologous CAR T-cells (15)

## 2 Risk factors for relapse

Mortality post relapse was found to be dependent on the timing of relapse with those less than 6 months from allo-HCT having worse outcomes than those relapsing between 6 months and 1 year and lower mortality if relapse occurs after 1 year from transplant (16).

The most common cause of transplant failure in ALL is relapse post allo-HCT which was particularly high with the presence of Ph chromosome. MRD status is also considered a key factor to predict relapse post-transplant and is defined as the presence of 0.01% or more detectable leukemia cells (17, 18). In the era of MRD prognostication, the pre-treatment cytogenetic information remains of value in the stratification of ALL (19, 20). Complex cytogenetics (≥ 5 chromosomal aberrations), and low hypodiploidy/near triploidy are independent predictive factors for worse survival outcomes (19). In the largest study by the Medical Research Council (MRC) UKALLXII/Eastern Cooperative Oncology Group (ECOG), patients harboring Ph, t(4;11)(q21;q23), t(8;14)(q24.1;q32), complex cytogenetics or low hypodiploidy/near triploidy, all had inferior rates of event-free and overall survival (OS) when compared with other patients (21). ALL with *KMT2A* rearrangement also has a poor prognosis regardless of the gene partner (22). In addition to the cytogenetic prognostic value, some genetic mutations were identified as independent predictors of survival including focal *IKZF1* gene deletion in B-ALL and the absence of *NOTCH1/FBXW7* mutation and/or *N/K-RAS* mutation and/or *PTEN* gene alteration in T-cell ALL (23). Ph-like ALL is another entity that is associated with lower rates of response and poor survival, especially those carrying *CRLF2* rearrangement and *IKZF1* deletion (24).

Several mutations are acquired at relapse of ALL affecting mainly five different molecular pathways including purine metabolism, histone modification, RAS signaling, tyrosine kinase signaling, and progenitor cell development (25). Gene expression profiling of blasts in ALL patients post relapse revealed the presence of a common genetic profile with upregulation of genes involved in proliferation and cell regulation such as *DUSP6*, *UBE2V1* and *F2R*, together with genes promoting apoptosis inhibition as BIRC5 (26). Moreover, DNA repair genes such as *PTTG1* and *UBE2V1* were negatively affected besides the presence of genes related to drug resistance such as *TYMS* and *RAB5C* (25). Epigenetic mechanisms play an essential role in the emergence of relapsed disease where significant differences in the CpG sites of DNA methylation were detected in blasts at diagnosis compared to those at relapse, indicating a change in the genomic expression (27).

## 3 Prevention of relapse

### 3.1 Established strategies

#### 3.1.1 The role of tyrosine kinase inhibitors

One of the most common cytogenetic abnormalities in adult ALL is the presence of Ph that is established as an adverse prognostic marker. The incorporation of TKIs in the frontline treatment of Ph-positive ALL followed by allo-HCT in first CR has shown improvement in long-term survival (1). Despite the progress in allo-HCT techniques in Ph-positive ALL and the substantial improvement in post-transplant relapse therapies with a steady increase in 2-year OS over the years from 28% to 55%, leukemia relapse remains the main cause of failure post allo-HCT (28). Thus, maintenance therapies with TKI were developed to mitigate the risk of relapse. Nowadays, the use of TKIs after allo-HCT is encouraged for all patients with Ph-positive ALL irrespective of the MRD status (prophylactic approach) once hematopoietic stability is achieved, or at least preemptively upon MRD recurrence. In a prospective randomized trial, prophylactic use of imatinib post allo-HCT showed a lower incidence of molecular relapse compared to MRD driven imatinib use (40% versus 69%, p=0.046), with a relatively high 5-year OS of 80% and 74.5% respectively, despite early discontinuation of imatinib due to adverse events (10). Imatinib was used prophylactically or preemptively post-transplant in seven prospective studies and five retrospective studies showing a 1.5 to 3 years OS ranging between 62% to 92% and 1.5 to 5 years disease free survival of 60.4% to 92%. On the other hand, second generation TKIs such as dasatinib and nilotinib were studied post allo-HCT with limited data suggesting better OS with second-generation TKIs especially in MRD positive patients. Dasatinib maintenance led to an OS of 87% to 100% at 1.4 to 3 years and disease-free survival was 89% to 100% which is higher than that with imatinib. In a study using nilotinib in Ph positive ALL, OS at 5 years was only 60% (29, 30). Furthermore, up to 50% of relapses post allo-HCT are caused by the BCR-ABL T315I mutation. Thus, prophylactic or pre-emptive use of ponatinib in this setting was tested in retrospective studies showing good tolerability of 15 mg daily dosing, and efficacy in molecular response as well as long-term OS and leukemia-free survival (31, 32). The use of TKIs (from all generations) after allo-HCT for patients in first CR improved OS when given as a prophylactic or preemptive regimen (33). In addition, imatinib failed to improve OS in patients who were beyond first CR at the time of allo-HCT and no data were available with newer generation TKIs for this population (33).

In a retrospective study conducted by the European Society for Blood and Marrow Transplantation (EBMT), the use of a prophylactic TKI in 60 patients post allo-HCT was associated with improved leukemia-free survival (hazard ratio [HR]=0.44, p=0.002) and OS (HR=0.42, p=0.004) (34). Based on this and many other published reports, the EBMT published a consensus statement encouraging the use of maintenance TKI post allo-HCT to reduce risk of relapse (35).

The duration of TKI maintenance post allo-HCT is another debate. There are no current prospective trials evaluating the optimal duration of TKI maintenance. Institutional guidelines recommend continuing TKI for at least 2 years and up to 5 years, if no major toxicities arise. In the GMALL study, imatinib maintenance was given for one year of continuous complete molecular remission (10). A recent retrospective study from MD Anderson cancer center evaluated 84 Ph-positive ALL patients who were alive and in complete molecular remission at 3 months post allo-HCT and still receiving TKI. The median duration of TKI maintenance was 13 months. Patients who received TKI beyond 24 months had significantly lower risk of relapse than those who stopped before 24 months (HR=0.12, p=0.045) (36). Nevertheless, prolonged administration of TKI post allo-HCT may increase drug-related toxicities over time affecting drug tolerability and compliance.

#### 3.1.2 The role of donor lymphocyte infusion

Donor lymphocyte infusions (DLIs) are often used to treat leukemia relapse post allo-HCT by re-instituting the T-cell immunity against the leukemia cells. It is now known that DLI effectiveness is dependent on many factors, including the type of underlying disease, the bulk of disease, and the pre-treatment immunologic state, mainly CD8+ T-cell infiltrates (37). While DLI response rates are relatively high in chronic myeloid leukemia, reaching 80%, the use of therapeutic DLIs in relapsed B-cell ALL after allo-HCT is still disappointing with remissions not exceeding 15% (38–40). One of the proposed reasons for the relative ineffectiveness of therapeutic DLI in ALL is the lack of proliferative capacity of ALL-reactive T-cells (41). Considering the limited efficacy of DLI in relapsed B-ALL, several groups have shown that prophylactic or pre-emptive DLI based on the MRD status or the loss of complete chimerism may have a role in preventing overt hematological relapse by infusing multiple escalated doses of DLI guided by the occurrence of GVHD (11, 12, 42). This approach was proven to reduce the relapse risk and increase survival post allo-HCT in patients with impending relapse. In order to enhance the GVL effect while limiting the known risk of GVHD post DLI, a recent pilot study investigated a repetitive schedule of low-dose DLIs every two months for at least 36 months and included 11 patients with high-risk ALL post allo-HCT. This innovative strategy was demonstrated to be safe and effective in reducing both relapse and GVHD rates in patients with high-risk acute leukemia (43). An alternative strategy involves a dose escalation schedule of DLI that similarly provides improved outcomes with lower rates of GVHD (44). In addition, DLI can be combined with blinatumomab in the post-transplant setting, allowing the infused donor T-cells to be redirected toward the CD19-positive leukemia cells. This combination was tested in a few retrospective studies that showed its tolerability, however with no definite added benefit (45, 46).

### 3.2 Innovative strategies

#### 3.2.1 The role of blinatumomab

Blinatumomab is a recombinant murine monoclonal bispecific antibody that targets both CD19 antigen expressed by most B-cells and the CD3 antigen present on T cells leading to their engagement, activation of T cells and subsequent lysis of leukemia cells (47). Blinatumomab was granted the US Food and Drug Administration (FDA) approval in 2014 for adults and children with R/R B-ALL or those with persistent MRD positive ALL based on a multi-institutional randomized phase III trial that demonstrated superiority of blinatumomab over chemotherapy in regards to overall response rate (ORR) and OS in patients with R/R ALL (48). Despite the great enthusiasm, blinatumomab as a single agent in overt relapse offered a modest response rate of 40% and a median OS not exceeding 8 months (48). An exploratory analysis on 64 patients who had relapsed post allo-HCT and were treated with blinatumomab showed similar outcomes with an ORR of 45% and median OS of 8.5 months (49). More importantly, there were no increased rates of GVHD, nor infections. It was soon established that blinatumomab is more effective when the burden of disease is low. The BLAST clinical trial investigated blinatumomab in 113 patients in first or second CR with detectable MRD ≥10^-3^, where 78% achieved a complete MRD response and 67% proceeded to allo-HCT (50). In patients with chemotherapy-resistant MRD, targeted immunotherapy with blinatumomab resulted in a substantial molecular response rate and improved long-term outcomes among responders, even those who were not transplanted (51).

To decrease the risk of relapse post-transplant by redirecting the unengaged donor T-cells before they mount a GVL response toward residual leukemic cells, Gaballa et al. investigated the use of maintenance blinatumomab post allo-HCT in high-risk B-ALL (13). In a phase II study conducted at The MD Anderson Cancer Center, four cycles of maintenance blinatumomab were administered every 3 months in the first year after allo-HCT. Patients at high risk of relapse other than those with persistent MRD pre and/or post allo-HCT were included such as complete hematologic remission beyond first CR at the time of allo-HCT, primary induction failure requiring more than one line of treatment, and/or high-risk cytogenetic or molecular profile defined as Ph-positive ALL, Ph-like ALL, *KMT2A* gene rearrangement, complex cytogenetics, or hypodiploid cytogenetics at diagnosis. Twenty-one patients were treated with at least one cycle of blinatumomab, and 57% completed all scheduled four cycles. The median follow-up was for 14.3 months with a cumulative incidence of relapse of 29% (95% confidence interval: 11%-49%). The 1-year OS and progression-free survival (PFS) were 85% and 71%, respectively (13). As reported previously, blinatumomab was not associated with increased risk of GVHD nor infections. Interestingly, this study highlighted some important mechanistic insights on predictors of response including the number of CD4 and CD8 T-cells with effector memory phenotype and the expression of some checkpoint inhibitors especially TIM-3. Despite the established safety of blinatumomab post allo-HCT, its additional benefit was not confirmed when compared to a matched cohort not treated with blinatumomab. Furthermore, blinatumomab could have a preferential benefit in patients with genomic loss of human leucocyte antigen expression after haplo-identical HCT where three out of four patients achieved CR with complete MRD response after 2 cycles of treatment, suggesting blinatumomab therapy as a potential strategy to restore a GVL effect (52). The mentioned data indicate a remarkable potential for blinatumomab as a promising agent to maintain remission and manage relapse after allo-HCT with the need for more randomized trials to optimize the standards. Additionally, there is also a rationale for combining blinatumomab with other agents like checkpoint inhibitors or DLI to further activate donor T-cells and increase efficacy; however, this approach might be limited by a substantial increase in the risk of acute life threatening GVHD.

#### 3.2.2 The role of inotuzumab ozogamicin

Inotuzumab ozogamicin is a CD22 antibody drug conjugate bound to calicheamicin, a cytotoxic antitumor antibiotic, that was approved by the FDA in 2017 for the treatment of adult patients with R/R B-ALL based on the results of the INO-VATE trial (53). The trial included 326 R/R B-ALL patients and among those who achieved CR, the percentage with MRD negativity was significantly higher in the inotuzumab ozogamicin (78.4%) compared to the standard therapy group (28.1%) (54). In addition, consolidation with allo-HCT for patients who achieved CR in the inotuzumab group led to better OS besides other factors that improved survival such as best MRD status, hematological remission and the duration of first CR (55). The CR rate was also similar between patients with or without prior allo-HCT (76.5% versus 81.5%) (54). The main limitation with inotuzumab ozogamicin is hepatotoxicity and veno-occlusive disease in patients receiving allo-HCT, for which preventive measures should be considered such as the avoidance of double alkylators within the conditioning regimen and the limitation of number of cycles of inotuzumab to up to 2 cycles if allo-HCT is planned, in addition to a longer interval between the last dose and allo-HCT, preferably six to eight weeks (56). The use of inotuzumab ozogamicin as maintenance treatment post allo-HCT at lower doses of 0.3 and 0.4 mg/m^2^ in four 28-days cycles was studied in a phase I trial which included eight patients with high-risk ALL. Four patients completed the first year of post-transplant follow-up, low-dose inotuzumab ozogamicin was well tolerated with only thrombocytopenia as a dose-limiting toxicity (14). The absence of disease recurrence in this small cohort of high-risk ALL patients warrants further investigation.

#### 3.2.3 The role of cellular therapy

Chimeric antigen receptor (CAR) T-cell therapy has led to significant success in hematological malignancies especially in R/R B-ALL where autologous CD19 CAR T-cells have shown remarkable anti-leukemia effects and CR rates of 70 to 90% (57). This led to tisagenlecleucel being the first CAR T-cell therapy product to be approved by the FDA in 2017 followed by brexucabtagene autoleucel as a second autologous product for R/R B-ALL (58). CAR T-cell therapy in the R/R setting is usually limited by a relatively short disease-free survival, for which successful CAR T-cell therapy is commonly followed by allo-HCT. However, data on CAR T-cells for post-transplant relapse is limited to few studies where the source of CAR T-cells can be from the patient him/herself (autologous) or from the transplant donor (allogeneic) (59). Allogeneic CAR T-cells can be responsible for lethal GVHD in some preclinical studies. In addition, a retrospective study comparing autologous to allogeneic CAR T-cell therapy demonstrated the presence of chronic GVHD in patients who received the allogeneic form (60). Ding et al. conducted a study on twenty patients with R/R B-ALL after allo-HCT that showed satisfactory initial efficacy and safety of CAR T-cell therapy. Moreover, the incidence of acute GVHD was higher in patients who previously received haploidentical transplantation especially for those receiving allogeneic CAR T-cells (15).

A promising new product generated by the Center for Cell and Gene Therapy, Baylor College of Medicine, involved multiple leukemia antigen–specific T-cells that targeted the tumor-associated antigens PRAME, WT1, and survivin, which are frequently expressed on B-cell and T-cell ALL cells from all 15 donors of patients with ALL who were undergoing allo-HCT. Eleven of the 15 products were infused into patients within 6 months post-transplant. There was an increase in the frequency of T-cells responding to targeted tumor antigens that correlated with durable remissions. Interestingly, no cases of GVHD were encountered after the infusion of the products (61).

## 4 Central nervous system prophylaxis

The role of CNS prophylaxis post allo-HCT in B-cell ALL is still a controversial issue. Given the high risk of CNS relapse especially in patients with CNS involvement, many transplant centers have endorsed the use of intrathecal chemotherapy post allo-HCT for primary or secondary prophylaxis despite the lack of well-designed clinical trials (62).

## 5 Role of maintenance treatment in T-cell acute lymphoblastic leukemia

High-risk or R/R T-cell ALL is an aggressive disease with few available salvage options and usually carries a dismal outcome with only 10% of patients surviving at 5 years (63). Preclinical studies have shown that relapsed T-ALL is sensitive to BCL2 inhibition exerted by venetoclax which could be a potential therapeutic strategy in combination with chemotherapy or hypomethylating agents (64). In the clinical setting, robust data is scarce regarding post-transplant maintenance in patients diagnosed with T-ALL. Four cases with high-risk T-ALL were treated with maintenance 5-azacitidine and venetoclax in the post-transplant setting with encouraging results, as all of them remained in CR after a median follow-up of 15 months (65). A Chinese study evaluated the use of low dose decitabine as maintenance post-transplant in ALL, with impressive results in patients with T-ALL, as none of seven treated patients have relapsed (66). These encouraging results are worth exploring in a prospective clinical trial.

One of the most promising immunotherapeutic drug in T-cell ALL is daratumumab (monoclonal antibody that targets CD38) given the robust expression of CD38 on the surface of malignant T- blasts. Preclinical data on human xenografts models of T-ALL showed efficacy of daratumumab with a significant reduction in leukemia burden in 14 of 15 xenograft models (67, 68). Daratumumab has started to be used in advanced T-ALL without any therapeutic options, and efficacy was shown to be in patients with low burden of disease (targeting MRD-positive disease) (69, 70). Currently, NCT05289687 is evalutating its role in MRD positive T-ALL. Given potential efficacy of daratumumab in low burden T-ALL, it is worth exploring it as maintenance treatment post allo-HCT in T-ALL.

## 6 Conclusion

Allo-HCT continues to have a successful role in curing a significant proportion of patients with high-risk ALL. However, relapse post allo-HCT remains a major challenge that requires more randomized clinical trials to optimize conditioning regimens and preventive measures following transplant. Several strategies are currently being used and studied, ranging from immunotherapeutic and targeted agents to maintenance with TKIs and the use of cellular therapies (**Table 2**). Therefore, further studies should focus on the contribution of each strategy and that of combinations of agents in the prevention of relapse post-transplant.

**Table 2:** Recommendations regarding some maintenance strategies post-transplant in ALL.

Disease	Agent	Recommendation
Ph-positive ALL	Tyrosine kinase inhibitors Imatinib Dasatinib Ponatinib	Recommended Encouraged Investigational
Ph-negative ALL	Blinatumumab Inotuzumab ozogamicin CAR T-cell therapy	Implemented by some transplant centers Investigational Investigational
T-cell ALL	HMA +/- venetoclax	Investigational

## Author contributions

MH was responsible for the citation research, referencing and writing of the manuscript. NM and JE contributed to the writing and referencing. IA and AB provided the topic of discussion, initial foundational research and guidance, extensive content review and editing, and final approval for submission. All authors contributed to the article and approved the submitted version.

## References

[1] Abou Dalle I Jabbour E Short NJ Ravandi F. Treatment of philadelphia chromosome-positive acute lymphoblastic leukemia. Curr Treat options Oncol (2019) 20(1):4. doi: 10.1007/s11864-019-0603-z 30675645 PMC10231844

[2] Künz T Hauswirth AW Hetzenauer G Rudzki J Nachbaur D Steiner N. Changing landscape in the treatment of adult acute lymphoblastic leukemia (ALL). Cancers (Basel) (2022) 14(17). doi: 10.3390/cancers14174290 PMC9454969 36077822

[3] Kegyes D Jitaru C Ghiaur G Ciurea S Hoelzer D Tomuleasa C. Switching from salvage chemotherapy to immunotherapy in adult b-cell acute lymphoblastic leukemia. Blood Rev (2023), 101042. doi: 10.1016/j.blre.2023.101042 36732205

[4] Rijneveld AW van der Holt B SM D BJ B de Weerdt O Muus P. Intensified chemotherapy inspired by a pediatric regimen combined with allogeneic transplantation in adult patients with acute lymphoblastic leukemia up to the age of 40. Leukemia (2011) 25(11):1697–703. doi: 10.1038/leu.2011.141 21647160

[5] Abou Dalle I Jabbour E Short NJ. Evaluation and management of measurable residual disease in acute lymphoblastic leukemia. Ther Adv Hematol (2020) 11:2040620720910023. doi: 10.1177/2040620720910023 32215194 PMC7065280

[6] Arslan S Pullarkat V Aldoss I. Indications for allogeneic HCT in adults with acute lymphoblastic leukemia in first complete remission. Curr Treat Options Oncol (2021) 22(7):63. doi: 10.1007/s11864-021-00860-1 34097131

[7] Abou Dalle I El Cheikh J Bazarbachi A. Pharmacologic strategies for post-transplant maintenance in acute myeloid leukemia: It is time to consider! Cancers (Basel) (2022) 14(6). doi: 10.3390/cancers14061490 PMC8946578 35326641

[8] Bazarbachi A Bug G Baron F Brissot E Ciceri F Dalle IA. Clinical practice recommendation on hematopoietic stem cell transplantation for acute myeloid leukemia patients with FLT3-internal tandem duplication: a position statement from the acute leukemia working party of the european society for blood and marrow transplantation. Haematologica (2020) 105(6):1507–16. doi: 10.3324/haematol.2019.243410 PMC7271578 32241850

[9] Kreidieh F Abou Dalle I Moukalled N El-Cheikh J Brissot E Mohty M. Relapse after allogeneic hematopoietic stem cell transplantation in acute myeloid leukemia: an overview of prevention and treatment. Int J Hematol (2022) 116(3):330–40. doi: 10.1007/s12185-022-03416-7 35841458

[10] Pfeifer H Wassmann B Bethge W Dengler J Bornhäuser M Stadler M. Randomized comparison of prophylactic and minimal residual disease-triggered imatinib after allogeneic stem cell transplantation for BCR-ABL1-positive acute lymphoblastic leukemia. Leukemia (2013) 27(6):1254–62. doi: 10.1038/leu.2012.352 23212150

[11] Dominietto A Pozzi S Miglino M Albarracin F Piaggio G Bertolotti F. Donor lymphocyte infusions for the treatment of minimal residual disease in acute leukemia. Blood (2007) 109(11):5063–4. doi: 10.1182/blood-2007-02-072470 17522340

[12] Yan CH Liu DH Liu KY Xu LP Liu YR Chen H. Risk stratification-directed donor lymphocyte infusion could reduce relapse of standard-risk acute leukemia patients after allogeneic hematopoietic stem cell transplantation. Blood (2012) 119(14):3256–62. doi: 10.1182/blood-2011-09-380386 22337715

[13] Gaballa MR Banerjee P Milton DR Jiang X Ganesh C Khazal S. Blinatumomab maintenance after allogeneic hematopoietic cell transplantation for b-lineage acute lymphoblastic leukemia. Blood (2022) 139(12):1908–19. doi: 10.1182/blood.2021013290 PMC8952188 34914826

[14] Metheny L III Sobecks R Tomlinson BK Otegbeye F Cooper B Majhail NS. Inotuzumab ozogamicin post-transplant for acute lymphocytic leukemia. Blood (2019) 134(Supplement_1):1948–8. doi: 10.1182/blood-2019-125940

[15] Ding L Wang Y Hong R Zhao H Zhou L Wei G. Efficacy and safety of chimeric antigen receptor t cells in acute lymphoblastic leukemia with post-transplant relapse. Front Oncol (2021) 11. doi: 10.3389/fonc.2021.750218 PMC8591161 34790576

[16] Dahlberg A Leisenring W Bleakley M Meshinchi S Baker KS Summers C. Prognosis of relapse after hematopoietic cell transplant (HCT) for treatment of leukemia or myelodysplastic syndrome (MDS) in children. Bone Marrow Transplant (2019) 54(8):1337–45. doi: 10.1038/s41409-019-0438-z PMC6646113 30670822

[17] Campana D. Minimal residual disease in acute lymphoblastic leukemia. Hematol Am Soc Hematol Educ Program (2010) 2010(1):7–12. doi: 10.1182/asheducation-2010.1.7 21239764

[18] Ravandi F Jorgensen JL O'Brien SM Jabbour E Thomas DA Borthakur G. Minimal residual disease assessed by multi-parameter flow cytometry is highly prognostic in adult patients with acute lymphoblastic leukaemia. Br J haematology (2016) 172(3):392–400. doi: 10.1111/bjh.13834 PMC4826052 26492205

[19] Issa GC Kantarjian HM Yin CC Qiao W Ravandi F Thomas D. Prognostic impact of pretreatment cytogenetics in adult philadelphia chromosome-negative acute lymphoblastic leukemia in the era of minimal residual disease. Cancer (2017) 123(3):459–67. doi: 10.1002/cncr.30376 PMC5321542 27696391

[20] Wetzler M Dodge RK Mrózek K Carroll AJ Tantravahi R Block AW. Prospective karyotype analysis in adult acute lymphoblastic leukemia: the cancer and leukemia group b experience. Blood (1999) 93(11):3983–93. 10339508

[21] Moorman AV Harrison CJ Buck GA Richards SM Secker-Walker LM Martineau M. Karyotype is an independent prognostic factor in adult acute lymphoblastic leukemia (ALL): analysis of cytogenetic data from patients treated on the medical research council (MRC) UKALLXII/Eastern cooperative oncology group (ECOG) 2993 trial. Blood (2007) 109(8):3189–97. doi: 10.1182/blood-2006-10-051912 17170120

[22] Richard-Carpentier G Kantarjian HM Tang G Yin CC Khoury JD Issa GC. Outcomes of acute lymphoblastic leukemia with KMT2A (MLL) rearrangement: the MD anderson experience. Blood Adv (2021) 5(23):5415–9. doi: 10.1182/bloodadvances.2021004580 PMC9153023 34525185

[23] Beldjord K Chevret S Asnafi V Huguet F Boulland ML Leguay T. Oncogenetics and minimal residual disease are independent outcome predictors in adult patients with acute lymphoblastic leukemia. Blood (2014) 123(24):3739–49. doi: 10.1182/blood-2014-01-547695 24740809

[24] Jain N Roberts KG Jabbour E Patel K Eterovic AK Chen K. Ph-like acute lymphoblastic leukemia: a high-risk subtype in adults. Blood (2017) 129(5):572–81. doi: 10.1182/blood-2016-07-726588 PMC5290985 27919910

[25] Bhatla T Jones CL Meyer JA Vitanza NA Raetz EA Carroll WL. The biology of relapsed acute lymphoblastic leukemia: opportunities for therapeutic interventions. J Pediatr Hematol Oncol (2014) 36(6):413–8. doi: 10.1097/mph.0000000000000179 PMC4264573 24942023

[26] Bhojwani D Kang H Moskowitz NP Min DJ Lee H Potter JW. Biologic pathways associated with relapse in childhood acute lymphoblastic leukemia: a children's oncology group study. Blood (2006) 108(2):711–7. doi: 10.1182/blood-2006-02-002824 PMC1895482 16822902

[27] Hogan LE Meyer JA Yang J Wang J Wong N Yang W. Integrated genomic analysis of relapsed childhood acute lymphoblastic leukemia reveals therapeutic strategies. Blood (2011) 118(19):5218–26. doi: 10.1182/blood-2011-04-345595 PMC3217405 21921043

[28] Bazarbachi A Labopin M Aljurf M Niittyvuopio R Balsat M Blaise D. 20-year steady increase in survival of adult patients with relapsed philadelphia-positive acute lymphoblastic leukemia post allogeneic hematopoietic cell transplantation. Clin Cancer Res (2022) 28(5):1004–12. doi: 10.1158/1078-0432.Ccr-21-2675 35022319

[29] Maher KR McBride A Amaraneni A Okolo O Farooqui SR Anwer F. Post-allogeneic stem cell transplantation maintenance dasatinib in philadelphia chromosome positive acute leukemia. Biol Blood Marrow Transplant (2017) 23(3):S289. doi: 10.1016/j.bbmt.2016.12.201

[30] Shimoni A Volchek Y Koren-Michowitz M Varda-Bloom N Somech R Shem-Tov N. Phase 1/2 study of nilotinib prophylaxis after allogeneic stem cell transplantation in patients with advanced chronic myeloid leukemia or p hiladelphia chromosome–positive acute lymphoblastic leukemia. Cancer (2015) 121(6):863–71. 10.1002/cncr.29141 25387866

[31] Leotta S Markovic U Pirosa MC Stella S Tringali S Martino M. The role of ponatinib in adult BCR-ABL1 positive acute lymphoblastic leukemia after allogeneic transplantation: a real-life retrospective multicenter study. Ann Hematol (2021) 100(7):1743–53. doi: 10.1007/s00277-021-04504-0 33774681

[32] Chen H Xu LP Zhang XH Wang Y Chen YH Yan CH. Safety and outcomes of maintenance therapy with third-generation tyrosine kinase inhibitor after allogeneic hematopoietic cell transplantation in philadelphia chromosome positive acute lymphoblastic leukemia patients with T315I mutation. Leuk Res (2022) 121:106930. doi: 10.1016/j.leukres.2022.106930 36007342

[33] Warraich Z Tenneti P Thai T Hubben A Amin H McBride A. Relapse prevention with tyrosine kinase inhibitors after allogeneic transplantation for philadelphia chromosome–positive acute lymphoblast leukemia: A systematic review. Biol Blood Marrow Transplant (2020) 26(3):e55–64. doi: 10.1016/j.bbmt.2019.09.022 31557532

[34] Brissot E Labopin M Beckers MM Socié G Rambaldi A Volin L. Tyrosine kinase inhibitors improve long-term outcome of allogeneic hematopoietic stem cell transplantation for adult patients with philadelphia chromosome positive acute lymphoblastic leukemia. Haematologica (2015) 100(3):392–9. doi: 10.3324/haematol.2014.116954 PMC4349279 25527562

[35] Giebel S Czyz A Ottmann O Baron F Brissot E Ciceri F. Use of tyrosine kinase inhibitors to prevent relapse after allogeneic hematopoietic stem cell transplantation for patients with philadelphia chromosome-positive acute lymphoblastic leukemia: A position statement of the acute leukemia working party of the european society for blood and marrow transplantation. Cancer (2016) 122(19):2941–51. doi: 10.1002/cncr.30130 27309127

[36] Saini N Marin D Ledesma C Delgado R Rondon G Popat UR. Impact of TKIs post-allogeneic hematopoietic cell transplantation in philadelphia chromosome-positive ALL. Blood (2020) 136(15):1786–9. doi: 10.1182/blood.2019004685 PMC8209550 32492706

[37] Bachireddy P Wu CJ. Understanding anti-leukemia responses to donor lymphocyte infusion. Oncoimmunology (2014) 3:e28187. doi: 10.4161/onci.28187 25340010 PMC4203631

[38] Dazzi F Szydlo RM Cross NC Craddock C Kaeda J Kanfer E. Durability of responses following donor lymphocyte infusions for patients who relapse after allogeneic stem cell transplantation for chronic myeloid leukemia. Blood (2000) 96(8):2712–6. doi: 10.1182/blood.V96.8.2712 11023502

[39] Collins RH Jr. Goldstein S Giralt S Levine J Porter D Drobyski W. Donor leukocyte infusions in acute lymphocytic leukemia. Bone Marrow Transplant (2000) 26(5):511–6. doi: 10.1038/sj.bmt.1702555 11019840

[40] Collins RH Jr. Shpilberg O Drobyski WR Porter DL Giralt S Champlin R. Donor leukocyte infusions in 140 patients with relapsed malignancy after allogeneic bone marrow transplantation. J Clin Oncol (1997) 15(2):433–44. doi: 10.1200/jco.1997.15.2.433 9053463

[41] Nijmeijer BA van Schie ML Verzaal P Willemze R Falkenburg JH. Responses to donor lymphocyte infusion for acute lymphoblastic leukemia may be determined by both qualitative and quantitative limitations of antileukemic t-cell responses as observed in an animal model for human leukemia. Exp Hematol (2005) 33(10):1172–81. doi: 10.1016/j.exphem.2005.06.034 16219539

[42] Yan C-H Q-f L Wu D-P Zhang X Xu L-P Zhang X-H. Prophylactic donor lymphocyte infusion (DLI) followed by minimal residual disease and graft-versus-Host disease–guided multiple DLIs could improve outcomes after allogeneic hematopoietic stem cell transplantation in patients with Refractory/Relapsed acute leukemia. Biol Blood Marrow Transplant (2017) 23(8):1311–9. doi: 10.1016/j.bbmt.2017.04.028 28483716

[43] Tsirigotis P Gkirkas K Kitsiou V Chondropoulos S Athanassiades T Thomopoulos T. Repetitively administered low-dose donor lymphocyte infusion for prevention of relapse after allogeneic stem cell transplantation in patients with high-risk acute leukemia. Cancers (Basel) (2021) 13(11). doi: 10.3390/cancers13112699 PMC8198731 34070786

[44] Kothari S Artz AS Lee SM Fulton N Park JH Stock W. Dose escalation prophylactic donor lymphocyte infusion after t-cell depleted matched related donor allogeneic hematopoietic cell transplantation is feasible and results in higher donor chimerism, faster immune re-constitution, and prolonged progression-free survival. Bone Marrow Transplant (2020) 55(6):1161–8. doi: 10.1038/s41409-020-0798-4 31992847

[45] Durer C Durer S Shafqat M Shah Z Sadiq M Fraz MA. Concomitant use of blinatumomab and donor lymphocyte infusion for post-transplant relapsed CD19 positive acute lymphoblastic leukemia: Systematic review. Blood (2018) 132(Supplement 1):5742–2. doi: 10.1182/blood-2018-99-109998

[46] Chauvet P Paviglianiti A Labopin M Labussière H Boissel N Robin M. Combining blinatumomab and donor lymphocyte infusion in b-ALL patients relapsing after allogeneic hematopoietic cell transplantation: a study of the SFGM-TC. Bone Marrow Transplant (2023) 58(1):72–9. doi: 10.1038/s41409-022-01846-9 36261707

[47] Keating AK Gossai N Phillips CL Maloney K Campbell K Doan A. Reducing minimal residual disease with blinatumomab prior to HCT for pediatric patients with acute lymphoblastic leukemia. Blood Adv (2019) 3(13):1926–9. doi: 10.1182/bloodadvances.2018025726 PMC6616259 31243002

[48] Kantarjian H Stein A Gökbuget N Fielding AK Schuh AC Ribera JM. Blinatumomab versus chemotherapy for advanced acute lymphoblastic leukemia. N Engl J Med (2017) 376(9):836–47. doi: 10.1056/NEJMoa1609783 PMC5881572 28249141

[49] Stein AS Kantarjian H Gökbuget N Bargou R Litzow MR Rambaldi A. Blinatumomab for acute lymphoblastic leukemia relapse after allogeneic hematopoietic stem cell transplantation. Biol Blood Marrow Transplant (2019) 25(8):1498–504. doi: 10.1016/j.bbmt.2019.04.010 31002989

[50] Gökbuget N Dombret H Bonifacio M Reichle A Graux C Faul C. Blinatumomab for minimal residual disease in adults with b-cell precursor acute lymphoblastic leukemia. Blood (2018) 131(14):1522–31. doi: 10.1182/blood-2017-08-798322 PMC6027091 29358182

[51] Gökbuget N Zugmaier G Klinger M Kufer P Stelljes M Viardot A. Long-term relapse-free survival in a phase 2 study of blinatumomab for the treatment of patients with minimal residual disease in b-lineage acute lymphoblastic leukemia. Haematologica (2017) 102(4):e132–5. doi: 10.3324/haematol.2016.153957 PMC5395124 28082340

[52] Wu H Cai Z Shi J Luo Y Huang H Zhao Y. Blinatumomab for HLA loss relapse after haploidentical hematopoietic stem cell transplantation. Am J Cancer Res (2021) 11(6):3111–22. PMC8263683 34249448

[53] Rausch CR EJ J HM K Kadia TM. Optimizing the use of the hyperCVAD regimen: Clinical vignettes and practical management. Cancer (2020) 126(6):1152–60. doi: 10.1002/cncr.32606 31794095

[54] Kantarjian HM DeAngelo DJ Stelljes M Martinelli G Liedtke M Stock W. Inotuzumab ozogamicin versus standard therapy for acute lymphoblastic leukemia. New Engl J Med (2016) 375(8):740–53. doi: 10.1056/NEJMoa1509277 PMC5594743 27292104

[55] Komitopoulou A Baltadakis I Peristeri I Goussetis E. Immunotherapy and allogeneic bone marrow transplantation in b acute lymphoblastic leukemia: How to sequence? Clin Hematol Int (2022) 4(1):11–20. doi: 10.1007/s44228-022-00006-6 35950202 PMC9358786

[56] Jain T Litzow MR. Management of toxicities associated with novel immunotherapy agents in acute lymphoblastic leukemia. Ther Adv Hematol (2020) 11:2040620719899897. doi: 10.1177/2040620719899897 32010436 PMC6971963

[57] Turtle CJ Hanafi L-A Berger C Gooley TA Cherian S Hudecek M. CD19 CAR–t cells of defined CD4+: CD8+ composition in adult b cell ALL patients. J Clin Invest (2016) 126(6):2123–38. 10.1172/JCI85309 PMC4887159 27111235

[58] Ramos CA. Should CD19 CAR-t cells for ALL be followed by allogeneic stem cell transplant? Transplant Cell Ther (2022) 28(1):1–2. doi: 10.1016/j.jtct.2021.12.002 35067350

[59] Jacoby E Yang Y Qin H Chien CD Kochenderfer JN Fry TJ. Murine allogeneic CD19 CAR t cells harbor potent antileukemic activity but have the potential to mediate lethal GVHD. Blood J Am Soc Hematol (2016) 127(10):1361–70. doi: 10.1182/blood-2015-08-664250 PMC4786842 26660684

[60] Hu Y Wang J Wei G Yu J Luo Y Shi J. A retrospective comparison of allogenic and autologous chimeric antigen receptor t cell therapy targeting CD19 in patients with relapsed/refractory acute lymphoblastic leukemia. Bone Marrow Transplant (2019) 54(8):1208–17. doi: 10.1038/s41409-018-0403-2 30518980

[61] Naik S Vasileiou S Tzannou I Kuvalekar M Watanabe A Robertson C. Donor-derived multiple leukemia antigen-specific t-cell therapy to prevent relapse after transplant in patients with ALL. Blood (2022) 139(17):2706–11. doi: 10.1182/blood.2021014648 PMC9053698 35134127

[62] Sauter CS DeFilipp Z Inamoto Y Johnston L Nagler A Savani BN. ASBMT statement on routine prophylaxis for central nervous system recurrence of acute lymphoblastic leukemia following allogeneic hematopoietic cell transplantation. Biol Blood Marrow Transplant (2019) 25(3):e86–8. doi: 10.1016/j.bbmt.2018.12.757 30590125

[63] Baek DW Lee JM Kim J Cho HJ Moon JH Sohn SK. Therapeutic strategies, including allogeneic stem cell transplantation, to overcome relapsed/refractory adult t-cell acute lymphoblastic leukemia. Expert Rev Hematol (2021) 14(8):765–75. doi: 10.1080/17474086.2021.1960817 34313508

[64] Peirs S Matthijssens F Goossens S Van de Walle I Ruggero K de Bock CE. ABT-199 mediated inhibition of BCL-2 as a novel therapeutic strategy in t-cell acute lymphoblastic leukemia. Blood (2014) 124(25):3738–47. doi: 10.1182/blood-2014-05-574566 25301704

[65] Hassan MA Moukalled N El Cheikh J Bazarbachi A Abou Dalle I. Azacitidine in combination with venetoclax maintenance post-allogeneic hematopoietic stem cell transplantation in t cell acute lymphoblastic leukemia. Clin Hematol Int (2023) 5(1):52–5. doi: 10.1007/s44228-022-00019-1 PMC10063763 36595164

[66] Liu J Jiang ZX Xie XS Wan DM Cao WJ Wang M. Maintenance treatment with low-dose decitabine after allogeneic hematopoietic cell transplantation in patients with adult acute lymphoblastic leukemia. Front Oncol (2021) 11:710545. doi: 10.3389/fonc.2021.710545 34485147 PMC8415411

[67] Naik J Themeli M de Jong-Korlaar R Ruiter RWJ Poddighe PJ Yuan H. CD38 as a therapeutic target for adult acute myeloid leukemia and t-cell acute lymphoblastic leukemia. Haematologica (2019) 104(3):e100–3. doi: 10.3324/haematol.2018.192757 PMC6395314 30190344

[68] Bride KL Vincent TL Im SY Aplenc R Barrett DM Carroll WL. Preclinical efficacy of daratumumab in t-cell acute lymphoblastic leukemia. Blood (2018) 131(9):995–9. doi: 10.1182/blood-2017-07-794214 PMC5833263 29305553

[69] Ofran Y Ringelstein-Harlev S Slouzkey I Zuckerman T Yehudai-Ofir D Henig I. Daratumumab for eradication of minimal residual disease in high-risk advanced relapse of t-cell/CD19/CD22-negative acute lymphoblastic leukemia. Leukemia (2020) 34(1):293–5. doi: 10.1038/s41375-019-0548-z 31435023

[70] Cerrano M Bonifacio M Olivi M Curti A Malagola M Dargenio M. Daratumumab with or without chemotherapy in relapsed and refractory acute lymphoblastic leukemia. A retrospective observational Campus ALL study. Haematologica (2022) 107(4):996–9. doi: 10.3324/haematol.2021.279851 PMC8968887 35021604

